# Immunity to Fish Rhabdoviruses

**DOI:** 10.3390/v4010140

**Published:** 2012-01-18

**Authors:** Maureen K. Purcell, Kerry J. Laing, James R. Winton

**Affiliations:** 1 Western Fisheries Research Center, United States Geological Survey, 6505 NE 65th St., Seattle, WA 98115, USA; Email: jwinton@usgs.gov; 2 Department of Medicine, University of Washington, 1100 Fairview Ave. N., Seattle, WA 98109, USA; Email: klaing@fhcrc.org

**Keywords:** novirhabdovirus, interferon, cell-mediated immunity, neutralizing antibody, immune evasion, non-virion, apoptosis, persistent infections, host-cell shutoff

## Abstract

Members of the family Rhabdoviridae are single-stranded RNA viruses and globally important pathogens of wild and cultured fish and thus relatively well studied in their respective hosts or other model systems. Here, we review the protective immune mechanisms that fish mount in response to rhabdovirus infections. Teleost fish possess the principal components of innate and adaptive immunity found in other vertebrates. Neutralizing antibodies are critical for long-term protection from fish rhabdoviruses, but several studies also indicate a role for cell-mediated immunity. Survival of acute rhabdoviral infection is also dependent on innate immunity, particularly the interferon (IFN) system that is rapidly induced in response to infection. Paradoxically, rhabdoviruses are sensitive to the effects of IFN but virulent rhabdoviruses can continue to replicate owing to the abilities of the matrix (M) protein to mediate host-cell shutoff and the non‑virion (NV) protein to subvert programmed cell death and suppress functional IFN. While many basic features of the fish immune response to rhabdovirus infections are becoming better understood, much less is known about how factors in the environment affect the ecology of rhabdovirus infections in natural populations of aquatic animals.

## 1. Introduction

Rhabdoviruses are single-stranded RNA viruses and important pathogens of both wild and cultured fish throughout North America, Asia, and Europe [1]. Fish are hosts to a number of rhabdovirus species including: Infectious hematopoietic necrosis virus (IHNV), Viral hemorrhagic septicemia virus (VHSV), Hirame rhabdovirus (HIRRV), Snakehead rhabdovirus (SHRV) Spring viremia of carp virus (SVCV), Pike fry rhabdovirus (PRV), Starry flounder virus, and Ulcerative disease rhabdovirus (UDRV) [2,3]. Several of these viruses (IHNV, VHSV, and SVCV) are reportable to The World Organization for Animal Health (OIE). IHN primarily occurs in salmon and trout [4] and SVC primarily afflicts cyprinid fishes [5], while the list of VHS susceptible hosts continues to grow [6]. These viruses cause significant mortality and morbidity in both wild and cultured fish [1]. Consequently, many rhabdoviruses are well studied in their select fish hosts and the main mechanisms of protection of fish against rhabdoviruses are becoming clear. More detailed understanding of fish‑rhabdovirus interactions will continue to be elucidated as our knowledge of the teleost immune system expands. 

In this review, we discuss the general characteristics of fish rhabdoviruses and the factors contributing to their pathogenesis. Next, we will consider each major arm of the immune system in the context of fish rhabdoviral infection and will evaluate how rhabdoviruses evade or circumvent host defenses. Although we primarily focus on fish rhabdoviruses, at times insight gained from two closely related mammalian rhabdoviruses—Vesicular stomatitis virus (VSV) and Rabies virus (RABV)—will be drawn for comparison. Throughout the review, we will identify research gaps that, if answered, would enable a more comprehensive understanding of immunity to fish rhabdoviruses. 

## 2. The Fish Rhabdoviruses

### 2.1. Taxonomy

The rhabdovirus family contains viruses that are related in terms of both morphological and genetic characteristics [7]. Collectively, this growing viral family can infect a vast array of hosts in both plant and animal phyla and comprises over 160 species classified among six genera (*Vesiculovirus*, *Lyssavirus*, *Ephemerovirus*, *Novirhabdovirus*, *Cytorhabdovirus*, and *Nucleorhabdovirus*) [7]. The best studied of all fish rhabdoviruses are IHNV, VHSV, HIRRV and SHRV, which are accepted as members of the genus *Novirhabdovirus* [2]. SVCV is currently classified within the genus *Vesiculovirus* (typified by VSV) [2], but this taxonomic grouping is likely to change. 

### 2.2. Structure

**Figure 1:**
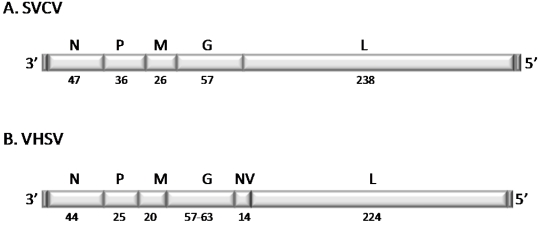
Genome organization and predicted molecular weights (MW) in kilodaltons of (**A**) a typical member of the genus *Vesiculovirus*, spring viremia of carp virus (SVCV) and (**B**) a typical member of the genus *Novirhabdovirus*, Viral hemorrhagic septicemia virus(VHSV). Predicted SVCV MW are based on [9] and for VHSV on [11].

Rhabdoviruses are bullet-shaped, enveloped viruses with a simple negative-sense, single-stranded RNA (ssRNA) genome [8]. The typical rhabdoviral genome encodes five basic structural proteins, including the nucleoprotein (N), phosphoprotein (P), matrix protein (M), glycoprotein (G) and the large polymerase (L) protein (Figure 1A) [9]. Additionally, all rhabdoviruses possess non-coding 3' leader and 5' trailer sequences. Members of the genus *Novirhabdovirus* are distinguished by the presence a sixth gene located in the genome between the G and the L genes (Figure 1B) encoding a non-structural (‘non-virion’ or NV) protein [10,11] that has a role in pathogenesis as discussed below. 

### 2.3. Infection Cycle

Understanding the viral infection cycle is important to define where the host immune system encounters virus-associated molecules. The general rhabdovirus life cycle, derived from VSV, has been reviewed extensively elsewhere and involves the five structural proteins found in all rhabdoviruses [8]. Briefly, the rhabdovirus enters its target cell by receptor-mediated endocytosis, which is triggered following engagement of a cell surface receptor by the viral G protein. The viral and endosomal membranes subsequently fuse and the viral nucleocapsid is released into the cytoplasm of the host cell. Within the cytoplasm, the viral genes are sequentially transcribed from the genome using an RNA-directed RNA polymerase that accompanies the infecting virion, viral proteins are synthesized by host cell machinery, and new copies of the genome are synthesized from a full-length, single‑stranded RNA anti-genome. To package the new virions, the N, L and P proteins, synthesized by free ribosomes in the cell cytoplasm, bind to the newly synthesized copies of the viral RNA genome to form the ribonucleoprotein (RNP) core, which associates with the M protein to produce the RNP-M complex. The G protein is synthesized by endoplasmic reticulum (ER)-bound ribosomes. It is glycosylated and further modified within the ER and Golgi apparatus prior to transport and insertion into the plasma membranes on the host cell surface. The RNP-M complex migrates to regions of the plasma membrane enriched with viral G proteins. The G protein studded host cell plasma membrane is subsequently captured by RNP-M protein complex as it buds from the cell to create fully enveloped rhabdovirus. 

### 2.4. Disease Manifestations

Viruses generally follow one of two strategies to ensure survival and transmission: the ‘hit and run’ or the ‘hit and stay’ [12]. As acute cytolytic viruses that cause high mortality, fish rhabdoviruses are typically considered ‘hit and run’ viruses. Their associated diseases are commonly characterized as acute hemorrhagic septicemias that impact multiple organs and present common clinical signs such as skin darkening, ascites, and exopthalmia [1]. However, varied forms of disease have been described for both IHN (hematopoietic and neurotropic) and VHS (acute, chronic and nervous) in rainbow trout (*Oncorhynchus mykiss*) [13,14], and a persistent infection/asymptomatic carrier state for both VHSV and IHNV has been reported that is often, but not always, associated with neural tissues [15,16,17,18,19]. There has been relatively little study of these alternative disease manifestations since most laboratory studies employ acute challenge models with virus doses sufficient to give reproducibly high mortality. 

### 2.5. Vaccination Strategies

In general, survivors of acute fish rhabdoviral infections develop long-lasting immunity, which is broadly protective against multiple virus strains of the same species [20,21]. In mammals, long-lasting immunity and anamnesis are characteristics of the adaptive immune response whereby memory T and B lymphocytes are developed during the initial infection by a pathogen. These cells then maintain surveillance for that same pathogen, perhaps for the life of the host, and are able to respond rapidly in the event of subsequent infection [22]. Vaccines exploit this characteristic by eliciting an anamnestic response prior to initial exposure to a pathogen, thus protecting the host against future infection. A variety of approaches have been investigated for vaccinating fish against rhabdoviral infections including attenuated strains, inactivated virus, subunit vaccines, and DNA vaccination [23,24,25,26]. These approaches are all efficacious to a degree (reviewed by [23,24,27]) but—with the exception of the IHNV DNA vaccine in Canada [28]—have not been licensed due to concerns regarding safety, consistency or cost. To overcome some of the safety concerns associated with DNA vaccines (see [29]), new vector constructs that use fish inducible promoters and a suicide gene (the IHNV M protein) to limit long-term survival of vaccine DNA in the muscle tissues have been developed and show promise [30,31].

Experimental DNA vaccines based on the rhabdoviral G protein of IHNV, VHSV, HIRRV and SVCV have shown efficacy in a number of different fish host species [32,33,34,35,36]. These G protein vaccines have been widely used as tools to probe the mechanisms of protective immunity in fish (for review see [37,38]). They elicit an early, nonspecific immune response that cross-protects against other viruses but not bacteria, while specific immunity arises later [39,40,41]. The G protein is the protective antigen but only in its correct conformation (for review see [23,24]). Large-scale production of glycosylated and folded G proteins has been difficult, limiting their commercial potential as subunit vaccines but recent successes with insect-based systems may change this [42]. However, the major barrier that remains to commercial rhabdoviral vaccination is the ability to mass vaccinate a large number of highly susceptible small fish. All fish rhabdoviral vaccines developed to date require individual handling and are typically administered via intramuscular or intraperitoneal injection. Novel immersion or oral vaccination approaches (*i.e.*, [43]) may eventually provide a solution to this barrier (reviewed recently in [44]). 

## 3. Immune Response

### 3.1. Mammalian Paradigm of Anti-Rhabdoviral Immunity

Conserved pattern recognition receptors (PRRs) of the innate immune system, such as the Toll-like receptor (TLR) and RIG-like receptor (RLR) families, detect ‘pathogen associated molecular patterns’ (PAMPs) and signal through conserved pathways to activate innate immune effector molecules [45]. In general, induction of anti-viral immunity follows recognition of viral nucleic acids by TLRs 3, 7 and 8 or the RLRs retinoic acid-inducible gene I (RIG-I) and melanoma differentiation-associated gene 5 (MDA5) and viral glycoproteins by TLRs [46]. This paradigm holds true for rhabdoviruses. For instance, engagement of both TLR7 by ssRNA and TLR4 by the viral G protein is required to mount an effective immune response against VSV in mice [47,48,49]. Both RIG-I and MDA5 are required for immunity against RABV [50] while RIG-I is also implicated in sensing VSV [51]. 

Signaling through PRRs leads to the activation of immune effector molecules, particularly the interferon (IFN) system. The IFNs mediate the primary early response to viruses. IFNs are powerful set of pleiotropic cytokines with diverse roles in regulating the immune system and inducing an anti‑viral state. They are divided into three major classes of gene families in mammals: Type I (IFN-α, β, ε, κ, τ and ω), Type II (IFN-γ) and Type III (IFN-λ) [52,53]. Type I IFNs are produced by a variety of cell types while Type II IFN expression is restricted to immune cells. It is well-established that RABV and VSV cannot replicate in type I IFN-alerted cells indicating the importance of type I IFN activity in controlling rhabdoviruses (reviewed in [54]). Furthermore, IFN-γ has been shown to reduce infection of murine neurons and neuroblastoma cells by VSV, whereby neuronal nitric oxide synthase (nNOS) activity is enhanced by IFN-γ to elicit viral control via production of reactive nitric oxide [55,56].

The IFNs signal through the conserved JAK/STAT pathway and up-regulate the expression of >300 IFN stimulated genes (ISGs) [57,58], which establish the ‘anti-viral’ state [59]. Many of these ISGs have direct anti-viral function (*i.e.*, Mx [60]) while the functions of other ISGs remain unknown. The ISG protein kinase R (PKR) binds double-stranded RNA (dsRNA) to inhibit viral protein synthesis [61]. Furthermore, mice deficient in PKR are more susceptible to VSV infections confirming the importance of this PRR in controlling rhabdoviruses [61]. 

The adaptive immune system is also an important component of the anti-rhabdoviral immunity. This arm of the immune system employs two classes of lymphocytes—B cell and T cells—that very specifically recognize viral antigens. B cells are absolutely essential for survival to VSV infection in mice [62]. They secrete neutralizing antibodies directed against the viral G protein, and are highly protective against VSV. Due to the repetitive, polyvalent nature of G protein epitopes on VSV virions—a typical characteristic of T cell independent antigens—these neutralizing antibodies are initially (days 2–6) produced in a T cell independent manner and represent predominantly the IgM isotype. Their secretion later becomes T cell dependent generating long-lived antibodies from both the IgM and IgG isotypes [63]. In mammals, there are two major T cell subsets: Cytotoxic T lymphocytes (CTL) that typically utilize the CD8 co-receptor and helper T cells (T_H_) that utilize the CD4 co‑receptor [22]. Studies in immune deficient mice highlight the requirement of CD4+ T cells for an optimal immune response against VSV and show that while CD4+ T cells can compensate for the their absence, CD8+ T cells also have anti-VSV activity [62]. In mammals, CTLs are responsible for specific cell-mediated cytotoxicity (CMC) while natural killer (NK) cells of the innate immune system are the primary effectors of nonspecific CMC [22]. Mammalian NK cells participate in the early innate antiviral immune response through non-antigen specific CMC of infected targets and secretion of IFN‑γ, while the CTL response occurs at later time point, and is MHC class I-restricted and antigen‑specific.

### 3.2. Viral Recognition in Fish

Pattern recognition of rhabdoviruses in fish is likely to mirror that observed in mammals [64]. Although there is limited functional characterization of receptor binding activity and signaling, teleost fish are known to possess the major TLR families plus an expanded set of unique non-mammalian TLR genes and gene variants (reviewed by [65,66]), as well as genes encoding RIG-I, MDA5, laboratory of genetics and physiology 2 (LGP2) and the associated signaling molecule mitochondrial antiviral signaling (MAVS) protein [67,68,69]. Fish possess orthologs to typical anti-viral TLRs including TLR3, TLR7 and TLR8 [70,71,72], in addition to novel TLRs (TLR22) that appear to bind different forms of RNA [73], suggesting there is conservation in recognition of viral nucleic acids. In contrast, most fish species either lack TLR4 or possess highly divergent TLR4-like genes relative to mammalian TLR4 (e.g., zebrafish (*Danio rerio*)) [74]. Thus, the phenomenon of viral glycoprotein recognition ascribed for TLR4 in mammals is unlikely to be conserved in fish species. However, the G proteins of novirhabdoviruses are known to trigger the synthesis of IFN in rainbow trout cells suggesting that capacity of the innate immune system to recognize rhabdoviral glycoproteins is conserved [75,76]; although the PRR(s) involved in this interaction are currently undetermined. The IFN response to the G protein can be triggered in both immune [75] and non-immune cells [76] and IFN induction is dependent on a peptide region that contains a canonical integrin binding site [77]. Other PRRs such as RIG-I, MDA5 and LGP2 (and their associated adaptor MAVS) activate the IFN system when overexpressed in fish cells, which correlates with protection against rhabdoviral infection [69,78]. 

### 3.3. Innate Immune Response to Fish Rhabdoviruses

Characterization of the innate immune response to fish rhabdoviruses has accelerated due to the genomic information available for model and commercially-important fish species. Genes encoding critical cytokines, chemokines, and other innate effectors have been identified in fish including those encoding both type I IFNs (also called IFNφ) and type II IFNs (also called IFNγ) [79,80,81,82,83,84,85,86,87,88,89]. Fish type I IFNs (IFNφ) are split into two major groups, which differ in the number of conserved cysteine (C) residues (group I—2C and group II—4C) [83,84] and appear to bind different receptors [90,91]. In general, group I IFNs are expressed in a wide range of tissue types while group II IFNs are more abundant in hematopoietic-derived tissues and cells [84]. Multiple gene copies of IFNγ exist in fish species but these genes are not duplicated to the same extent as the type I IFNs (reviewed in [92]). The phylogenetic relationships among fish IFNs and the associated nomenclature are confusing and disputed; the review by Zou and Secombes [92] provides a useful table summarizing the fish IFNs identified to date.

Like their mammalian counterparts, replication of fish rhabdoviruses is inhibited by pre-activation of the IFN system regardless of the method used, including using IFN-containing supernatants [93,94], poly I:C [95], rhabdoviral G protein [39,40,75] or recombinant IFN [96,97]. Prior to the identification of the IFN genes themselves, activation of a functional IFN response was inferred by virus plaque reduction [98], transcription of the Mx gene or other ISGs (*i.e.*, [99,100,101] and many other studies), or activation of an Mx promoter-reporter assay [102]. Early studies clearly indicate functional IFN in sera derived from fish infected with VHSV [93] , IHNV [98] or SVCV [94]. Later studies confirmed these results and demonstrate transcriptional up-regulation of both group I and II IFN genes after infection with these same viruses [84,85,103]. IFNγ genes are also regulated early in rhabdoviral infection [85]. 

Rhabdoviral infections are controlled by fish group I and II IFNs. Recombinant group I IFNs inhibit rhabdoviral replication [84,96,97,103,104] while the effects of group II IFNs on rhabdoviruses vary (e.g., [84]). A recent study demonstrates that recombinant group II IFNs protect zebrafish against SVCV infection and induce a rapid and transient up-regulation of ISGs whereas those from group I up‑regulate both ISGs and pro-inflammatory cytokines in a more sustained fashion [103]. Similarly, fish IFNγ also elicits an immune response related to controlling viruses. Recombinant IFNγ up-regulates a number of ISGs [105] and displays anti-viral activity [106] in a variety of Atlantic salmon (*Salmo salar*) cell-lines. At least some of the anti-viral activity ascribed to IFNγ may be due to the ability of IFNγ to increase transcription of group I IFNs [106]. However, recombinant IFNγ does not improve survival of zebrafish infected with SVCV [103], questioning whether rhabdoviruses are directly controlled via the activity of this cytokine.

Rhabdoviral infection and/or G protein vaccination induce a characteristic transcriptional pattern dominated by ISGs that are conserved throughout vertebrates, as well as the expression of novel antiviral genes (for a comprehensive review see [107]). Vaccination with the rhabdoviral G protein primes the systemic up-regulation of these ISGs [108,109,110] and the timing of this response correlates with nonspecific protection from virulent IHNV and VHSV challenge [39,40]. Fish rhabdoviral infection also result in a rapid IFN and ISG response—similar to that observed early after G protein vaccination—which correlates with virus levels in the tissues, but does not necessarily correlate with protection [101,111,112,113]. This paradox is suggestive of a virus-host race in which rapid replication of the fish rhabdoviruses competes kinetically against mobilization of innate immune mediators. Rapid replication is a hallmark of highly virulent IHNV strains [114,115,116]. Rainbow trout progeny groups with greater genetic resistance to IHNV have lower *in vivo* viral replication starting at less than 24 h post-infection [117], which is a relatively limited time frame for the host to induce a response that relies on new protein synthesis (*i.e.*, the IFN system). This suggests that constitutive or other innate barriers to viral replication are also important for overall disease resistance. Other studies have found that VHSV resistance in trout is correlated with *ex vivo* replication of VHSV in fin tissues, implicating epidermal tissues as pivotal in the anti-VHS defense system [118,119,120]. Host-virus dynamics at virus entry points (*i.e.*, the fins [121]) may help limit the internal spread of viral infection by alerting systemic sites via IFNs and other cytokines. 

### 3.4. Humoral Immune Response to Fish Rhabdoviruses

Neutralizing antibodies induced by infection and/or vaccination are critical components of long-term adaptive immunity to fish rhabdoviruses (reviewed in [122]). Passive immunization with sera containing neutralizing antibodies protects recipient juvenile trout from IHN and Pacific herring from VHS, even when titers fall below detectable levels [40,123,124,125]. Most studies of fish rhabdoviruses have focused on the highly protective neutralizing antibody response but it is possible that non-neutralizing or other types of antibodies may also play a role. *In vitro* rhabdoviral neutralization is complement-dependent but the exact mechanism by which complement aids viral neutralization is still unclear [126]. Neutralizing antibodies are unlikely to play a role in surviving the acute infection phase in coldwater fish species since neutralizing antibodies typically are not detectable until several weeks post-infection. Study of attenuated or low virulence virus types indicates that a certain threshold of virus replication must occur for fish to develop a broadly protective antibody response, even if a robust innate response is induced [113,127]. 

Lorenzen and LaPatra [122] published a comprehensive review of the antibody response to fish rhabdoviruses in 1999, and since then, this area has not received much new attention. Certainly, studies of the G protein DNA vaccines over the past decade have reaffirmed the central role of neutralizing antibodies in protective immunity [33,34,128,129,130,131,132,133]. However, genomic approaches combined with functional studies have brought exciting new insights into B cell biology in teleost fish. Fish B cells show phagocytic behavior suggesting that fish B cells may also function as part of the innate immune system [134]. Furthermore, it is now known that teleost fish possess three or more immunoglobulin isotypes including IgM, IgD and IgT (IgT is also called IgZ in zebrafish) [135,136,137]. A recent study indicates that B cells expressing IgM respond to antigenic stimulation in systemic tissues while B cells expressing IgT are key to the mucosal immune response [138]. To date, there has been no characterization of IgT during fish rhabdoviral infections. However, tools such as monoclonal antibodies to fish IgM and IgT now exist [138,139], which will allow the characterization of mucosal immunity and its relationship to systemic protection against rhabdoviruses. Finally, there has been much progress in unraveling the complexity of the fish complement system [140], which may help to finally define which complement components contribute to virus neutralization. 

### 3.5. Cellular Immune Response to Fish Rhabdoviruses

Although DNA vaccination with the novirhabdoviral G protein triggers production of protective neutralizing (and perhaps other) antibodies, high levels of specific protection after DNA vaccination are also observed without detectable neutralizing antibodies [33,130] indicating a potential role for specific cellular immunity. Major advances have been made recently in our understanding of fish T cells (as reviewed in [141]). Teleost fish possess a wide range of T cell associated genes, including genes that encode T cell receptor chains and various other T cell associated co-receptors, co‑stimulatory molecules and cytokines [141]. Many of these genes show up-regulated expression following rhabdoviral infection and/or G protein DNA vaccination [110,142]. There is a limited understanding of the role T cells play during fish rhabdoviral infection with most work focusing on cell-mediated cytotoxicity (CMC) (discussed below). However, VHSV infection and DNA vaccination with the VHSV G protein induces clonal expansion of T cells, as shown by spectratype analysis of the complementarity-determining region 3 (CDR3) of the TCR-β chain [143,144], supporting a role of teleost T cells in controlling this virus. Interestingly, the dominant CDR3 profiles are the same for both VHSV infection and G protein DNA vaccination suggesting that important T cell epitopes are localized within the G protein. Future studies examining the role of T cells during rhabdoviral infection will benefit from new reagents that can discriminate among T cell subsets (reviewed in [141]), including monoclonal antibodies against the CTL surface marker CD8 [145]. 

Studies using rainbow trout, channel catfish (*Ictalurus punctatus*), common carp (*Cyprinus carpio*) and crucian carp (*Carassius carassius*) provide evidence for both specific and nonspecific CMC in fish (reviewed in [146,147]). Two different cell types are responsible for nonspecific CMC in channel catfish, the nonspecific cytotoxic cells (NCC) and NK-like cells [148,149,150]. Common carp neutrophils also possess nonspecific CMC activity, evidenced by spontaneous killing of human tumor cell lines [151]. Studies of mammals and other vertebrate species indicate that NK cell activity is mediated by many different receptor types that can be specific to an individual animal species or even to a cell lineage [152]. Candidate NK receptor families in fish include the novel immune type receptors (NITRs), novel Ig-like transcripts (NILTs), leukocyte immune type receptors (LITRs), and possibly others (reviewed in [152]). Genomic characterization of fish species has enabled the identification of these polymorphic and polygenic receptor families, but there has been limited validation that these receptors have a functional role in NK activity. 

Functional studies of specific CMC were made possible in fish following the development of isogenic fish and major histocompatibility complex (MHC) I matched cell lines for both crucian carp and rainbow trout [153,154,155]. Somamoto *et al.* [155] demonstrated that peripheral blood and kidney derived lymphocytes from crucian carp exhibit a specific CMC response to MHC-matched cells infected with Crucian carp hematopoietic necrosis virus (CHNV; an uncharacterized fish rhabdovirus). Similarly, peripheral blood lymphocytes from rainbow trout infected with VHSV exhibit significant CMC to MHC class I matched target cells infected with VHSV but not to VHSV infected xenogeneic targets [154]. However, rainbow trout survivors re-exposed to VHSV mounted a kinetically faster, VHSV-specific CMC response, as well as a nonspecific CMC response against VHSV infected xenogeneic targets (starting at 11 days post-infection). The delayed nonspecific CMC response in trout contradicts the mammalian paradigm where primary CMC is nonspecific and results from NK cell activity while later CMC is pathogen-specific due to clonal expansion and action of virus specific CTLs [22]. Taken together, these studies suggest a role for cellular immunity—particularly CMC—in the host response to fish rhabdoviruses. However, more basic research is needed to definitely identify the cells that contribute to key cellular effector functions in fish.

### 3.6. Modulation of Immunity by Environmental Parameters

For poikilothermic vertebrates, temperature is a critical factor in host-pathogen interactions because most aspects of the host’s physiology, including the strength and speed of the immune response, as well as the replication rate of the pathogen are temperature dependent [156]. Among fish rhabdoviruses, it is well known that the severity of VHS and SVC alters with changing temperatures in the spring and fall [157,158]. However, while the *in vitro* replication rate of fish rhabdoviruses increases with temperature to a maximum upper limit [158], the severity of disease is generally less at higher temperatures which may be due to the greater efficiency of the fish immune response [159,160]. As such, modulation of temperature has been explored as a basis for protection or vaccination of fish against rhabdovirus infections [161,162]. 

At optimal temperatures, fish surviving rhabdovirus infections develop a robust immune response and clear the virus below detectable levels [163]. In contrast, fish held at lower temperatures may rely more on innate immunity and have inhibited or delayed specific immune responses [156,164,165]. Low or cold water temperatures are also linked to development of persistent rhabdoviral infections [15,17]. For example, rainbow trout held at 4 °C had detectable VHSV in the brain for over 400 days post‑infection and displayed no clinical signs of VHS or detectable serum neutralizing antibody titers [17]. These studies support the hypothesis that rhabdoviral persistence is related to suppression of the adaptive immune response. However, it is not clear whether individuals become long-term carriers or if the virus persists by cycling in the population among naïve and/or convalescent hosts [18]. If individuals are truly persistently infected, it raises interesting questions of how normally acute cytolytic viruses modulate replication and limit cytopathology. 

Other environmental factors may also affect disease resistance in fish. Intriguingly, the CMC response in fish immunized with the N protein of VHSV was suppressed during winter months despite constant water temperature and light regimen; this result may indicate that endogenous biological variables (e.g., reproductive cycle) or other seasonal factors influence anti-viral immunity [166]. Resistance to fish rhabdovirus infections has also been shown to be modulated by diet [167,168]. Thus, in addition to temperature, other environmental factors such as age, diet, seasonality and reproductive status will be important areas for study in order to fully understand the immune response to fish rhabdoviruses. 

## 4. Rhabdoviral Immune Evasion Mechanisms

### 4.1. Overview

A pathogenic virus must possess some mechanism to evade or limit innate host defenses, disrupt normal cellular processes, and gain preferential transcription and translation of its own genes [169]. Viruses have evolved many strategies to overcome host defenses. In response, the vertebrate immune system continuously evolves counter-strategies against the virus. This co-evolutionary arms race has led to great diversity in the host-virus relationship as viruses evolve new strategies to limit, evade or otherwise subvert the immune system. Here we will outline important aspects of immune evasion by mammalian rhabdoviruses. Next, we will consider evidence indicating a critical role for the fish rhabdoviral M and NV proteins in immune evasion. 

### 4.2. Mammalian Rhabdoviral Immune Evasion

As mentioned above, rhabdoviruses cannot replicate in IFN-primed cells (reviewed in [54]). However, the rapid and strong induction of the host IFN system following detection of invading rhabdoviruses is not sufficient to prevent the replication of virulent rhabdoviruses. Rhabdoviruses are thought to directly interfere with critical immune effector functions including the interferon system to evade immune control. They may also interfere with programmed cell death processes (apoptosis) and mediate global inhibition of host cellular gene expression and thereby inhibit transcription-dependent host cell defenses, a phenomenon known as host-cell shutoff [170]. RABV and VSV use very different strategies to disrupt mammalian host defenses. RABV relies on the P protein to limit IFN production, antagonize IFN signaling and directly interfere with IFN-induced antiviral molecules [171,172]. In contrast, VSV relies on the M protein to facilitate host-cell shutoff [170]. This mechanism limits RNA transcription by suppressing all host RNA polymerases [170], inhibits nucleocytoplasmic transport by blocking nuclear pore components [173], and prevents translation of host genes into proteins [174]. Shutoff of host cellular processes likely increases the resources available for viral transcription and translation, while decreasing production of anti-viral host proteins. 

Rhabdoviral infection induces apoptosis in infected mammalian cells (reviewed in [169]). Apoptosis is a type of programmed cell death that creates characteristic morphological and biochemical changes and is mediated by proteolytic enzymes called caspases [175]. In infection‑associated apoptosis, there arises a common question concerning whether the cell death is due to viral factors or to the host antiviral response. Both factors may be responsible. For instance, reverse genetic analysis of VSV revealed that apoptosis occurs by two distinct pathways; one pathway is associated with the M protein while the other pathway relies on host gene expression [176,177]. The M protein-mediated apoptosis may be a byproduct of the host-cell shutoff activity, since the suppression of host cellular gene expression and translation is most likely incompatible with long term cell survival. This hypothesis is supported by the finding that host-cell shutoff and apoptosis are genetically correlated and involve the mitochondrial (intrinsic) pathway of apoptosis and caspase 9 [176,177]. The second pathway is hypothesized to result from a typical host anti-viral response and requires both new host gene expression and caspase 8 to occur [176,178]. However, rapid induction of apoptosis is not effective at inhibiting VSV replication in most cell types suggesting that this is not necessarily an effective anti-viral response [177,179]. 

Antigenic/epitope variation is another strategy used by certain viruses, bacteria and parasites to evade the immune system and escape from protective humoral and/or cellular immune responses. Among viruses, this strategy is best known as an important feature in the biology of Influenza virus or Human immunodeficiency virus [180]. For rhabdoviruses, evidence for immune selection by antibody escape has been shown in variant laboratory strains of both RABV and VSV whereby escape mutants lose detectable reactivity with monoclonal antibodies specific for neutralizing epitopes on the virus G protein [181,182]. Field isolates of VSV from endemic areas show some evidence of immune selection [183,184]. Nevertheless, while playing a role in virus evolution, this strategy does not appear to be a critical factor in the persistence or epidemiology of rhabdoviruses in that antigenic strains of RABV and VSV continue to circulate in endemic areas without sequential replacement. Moreover, immunity conferred by infection or vaccination with one strain is broadly protective and likely due to conservation of some neutralizing epitopes on the G proteins of rhabdoviruses [185]. 

### 4.3. Fish Rhabdoviral Immune Evasion: Role for M Protein

Showing similarity to VSV, the fish novirhabdovirus IHNV possesses host-cell shutoff ability that is specifically mediated by the M protein [186]. Transfection of cultured Chinook salmon embryonic cells (CHSE-214) with the IHNV M protein also induces host morphological changes consistent with apoptosis [186], indicating cell death can occur in the absence of live virus. In contrast, there is no observable molecular or cellular effect following transfection of the IHNV P protein [186]. To date, host cell-shutoff has only been documented for IHNV and not for the other fish rhabdoviruses. The availability of reverse genetic tools for IHNV, VHSV and SHRV [187,188,189] would allow further dissection of this response in fish, such as the development of M protein mutants deficient in host-cell shut off activity. 

### 4.4. Fish Rhabdoviral Immune Evasion: Role for NV Protein

Attempts to identify a function for the NV protein have led to contradictory results. Overexpressed IHNV NV causes cell rounding in CHSE-214 cells but not apoptosis leading to speculation that NV interacts with host cytoskeletal elements [186]. SHRV NV is dispensable for efficient replication in cultured fathead minnow cells (EPC cell line; [190,191]) and *in vivo* virulence in zebrafish [188,192]. In contrast, the IHNV NV is essential for optimal growth in EPC cells and virulence in rainbow trout [193]; a similar observation was made using a VHSV NV knockout in the yellow perch (*Perca flavescens*) host [194]. Two new studies now indicate a role for NV in delaying apoptosis and suppressing the host IFN system [187,195]. 

Many viruses possess strategies to delay or prevent apoptosis to prolong viral replication. Recently, Ammayappan and Vakharia [187] demonstrated that the VHSV NV protein has the ability to delay the onset of apoptosis in cell culture. In their study, recombinant VHSV lacking NV induced apoptosis sooner than wild-type VHSV. The anti-apoptotic function could be recovered by the IHNV NV [187], suggesting the NV protein’s anti-apoptotic function may be conserved despite low amino acid similarity between the VHSV and IHNV NV proteins (~48%) [193]. Furthermore, VHSV infection induces the activity of the initiator caspases 3, 8, and 9 [187] implicating the involvement of multiple apoptotic pathways that may be induced by specific viral components (e.g., M protein) and/or by host components (e.g., mediated by IFN, PKR *etc.*). More studies are needed to determine which apoptotic pathway(s) are being targeted for delay by the VHSV NV protein. 

A second recent study indicates that the NV also functions to limit the host IFN response in fish. Choi *et al.* [195] demonstrated that recombinant IHNV lacking the NV gene induced greater transcription of the rainbow trout Mx and IFN1 in RTG-2 cells and had higher levels of functional IFN, as measured by an Mx reporter assay [102]. The study demonstrated that the NV protein is actively transported to the nucleus and that nuclear transport and anti-IFN activity were dependent on a unique nuclear localization signal (NLS). Although the NLS was conserved among all IHNV strains examined, it was not conserved in VHSV indicating that further study is needed to determine if the NV from all novirhabdoviruses function in a similar manner. Furthermore, it would be interesting to determine if the anti-apoptotic activity of the NV protein (described above for VHSV) is genetically correlated with the ability to limit IFN and is dependent on nuclear localization. 

### 4.5. Limited Evidence for Antigenic Escape

Analogous to RABV and VSV, isolates of fish rhabdoviruses show a level of antigenic diversity [4,5,157,196], and respond to immune selection with monoclonal antibodies by generating escape mutants [197]. Analysis of the amino acid sequences encoded by the G protein gene of a diverse set of field isolates of IHNV showed that the maximum diversity occurred in the central region of the protein that contained many of the antigenic determinants [198] suggesting that immune selection may be helping to drive virus evolution. This central region of the G gene was used by Kurath *et al.* [199] to show that, compared to stocks of wild anadromous salmonids in their native range, genetic diversity was highest among trout populations reared in aquaculture where a high rate of virus replication/infection and the presence of large numbers of recovering/recovered animals would be expected to drive a greater level of immune selection. Comparison of the nonsynonymous and synonymous substitutions present in the G gene sequences obtained from cultured rainbow trout IHNV isolates suggests the possibility that some specific amino acids may be under positive selection [200]. Furthermore, this phylogenetic analysis revealed that isolates of IHNV from trout aquaculture appeared to be undergoing a higher rate of evolution with the most recent isolates positioned at branch tips, unlike isolates from wild populations [199]. However, within the range of genetic strains or serological variants that comprise a given species of fish rhabdovirus, laboratory studies show broad protective immunity in fish that recover from infection with a given strain of the virus or are vaccinated using antigens from a specific strain [20,21]. Thus, while these data indicate that immune selection may be an important driver of genetic diversity and virus evolution, fish rhabdoviruses appear to be incapable of undergoing complete antigenic escape, perhaps due to the presence of a functionally essential, and thus highly conserved, regions of the G protein. However, more study is needed of the immunological responses to alternate disease manifestations, such as persistent infections, to determine how the virus evades the host antibody response. 

## 5. Future Directions

Interactions between the immune system and rhabdoviruses are well studied for certain fish rhabdoviruses in certain hosts (*i.e.*, IHNV and VHSV in salmonid species), but there are still gaps in our knowledge. Over the past decade, much advancement has been made in understanding the genomic architecture of the fish immune system, which will facilitate future functional studies to fill in these gaps. There has also been progress in identifying the viral determinants of immune evasion for VHSV and IHNV using reverse genetics, but it is not known if these strategies are broadly conserved among all members of the genus *Novirhabdovirus.* Additionally, more work is needed to better define the detailed mechanisms underlying these evasion strategies. The knowledge gaps are even greater for the SVCV and other vesiculo-like viruses. It is likely that the M protein-mediated host-cell shutoff is conserved in SVCV, but other novel mechanisms may also exist. For probing some of these questions, zebrafish may serve as an excellent model because many tools are available to genetically manipulate this fish species. However, there should be some caution with relying exclusively on model fish species as some aspects of the host-pathogen relationship may not be represented during infections of non-natural hosts. 

How highly virulent fish rhabdoviruses persist in populations is a long unanswered question. The answer will likely vary depending on many factors, including the rhabdoviral species, host species, host life history, types of vectors, environment, and degree of anthropogenic manipulation. However, understanding how fish rhabdoviruses interact with the host immune system can provide a new perspective on this question. Infection with fish rhabdoviruses is often acute and associated with high mortality; survivors typically clear the virus and develop broadly protective immunity to re-infection. However, if this scenario were always to occur, a reduction in the number of susceptible hosts through mortality and increased herd immunity among recovered animals should theoretically drive the virus out of any natural population. There are several alternatives to this scenario that include, but are not limited to: (1) ‘immune’ fish can replicate the virus to sufficient levels for viral shedding and transmission to occur, (2) endogenous or exogenous parameters suppress fish immunity such that survivors can be re-infected, (3) fish rhabdoviruses have a broad enough host range that susceptible hosts are always available, or (4) these normally acute viruses can also persistently infect a small number of individuals to ensure survival when there are no susceptible hosts. Although there are a number of studies indicating that fish rhabdoviruses can establish persistent infection (described earlier), there remains a possibility that the perceived persistence in these studies reflects infection cycling among previously exposed individuals or in non-natural conditions. Distinguishing among these hypothesized scenarios will require the merging of immunology and epidemiology, which may be the key to unraveling the disease ecology of fish rhabdoviruses. 
